# High-efficiency electromagnetic energy harvesting using double-elliptical metasurface resonators

**DOI:** 10.1371/journal.pone.0291354

**Published:** 2023-12-21

**Authors:** Abdulrahman Ahmed Ghaleb Amer, Nurmiza Othman, Syarfa Zahirah Sapuan, Arokiaswami Alphones, Ali Ahmed Salem

**Affiliations:** 1 Faculty of Electrical and Electronic Engineering, Universiti Tun Hussein Onn Malaysia, UTHM, Batu Pahat, Johor, Malaysia; 2 School of Electrical and Electronic Engineering, Nanyang Technological University, Singapore, Singapore; 3 School of Electrical Engineering, Universiti Teknologi MARA (UITM), Shah Alam, Selangor, Malaysia; 4 Faculty of Electrical Engineering, Sana’a University, Sana’a, Yemen; Edinburgh Napier University, UNITED KINGDOM

## Abstract

This study introduces a metasurface (MS) based electrically small resonator for ambient electromagnetic (EM) energy harvesting. It is an array of novel resonators comprising double-elliptical cylinders. The harvester’s input impedance is designed to match free space, allowing incident EM power to be efficiently absorbed and then maximally channelled to a single load through optimally positioned vias. Unlike the previous research works where each array resonator was connected to a single load, in this work, the received power by all array resonators is channelled to a single load maximizing the power efficiency. The performance of the MS unit cell, when treated as an infinite structure, is examined concerning its absorption and harvesting efficiency. The numerical results demonstrate that the MS unit cell can absorb EM power, with near-perfect absorption of 90% in the frequency range of 5.14 GHz to 5.5 GHz under normal incidence and with a fractional bandwidth of 21%. The MS unit cell also achieves higher harvesting efficiency at various incident angles up to 60^*o*^. The design and analysis of an array of 4x4 double elliptical cylinder MS resonators integrated with a corporate feed network are also presented. The corporate feed network connects all the array elements to a single load, maximizing harvesting efficiency. The simulation and measurement results reveal an overall radiation to AC efficiency of about 90%, making it a prime candidate for energy harvesting applications.

## 1. Introduction

With the rapid advancement of wireless communication systems, EM waves are now omnipresent. Although batteries are still commonly used to power wireless devices, they have limitations such as high cost and limited lifespan. Energy harvesting has gained attention as a solution to these limitations, enhancing the mobility and reliability of low-power wireless devices [1]. Energy harvesting in radio frequency (RF) and microwave regimes has interesting features such as low cost and compact size. Rectenna is the primary device that captures radiated EM power from ambient by the receiving antenna and converts it into direct current (DC) through a rectifier circuit [2]. Traditional antennas have been used to capture energy from the environment, but they have limitations, such as their large size and low efficiency [3,4].

Metamaterials are artificial structures or materials with unique properties, such as negative permittivity, permeability and refractive index. The MSs are defined as the two-dimensional counterparts of the metamaterials. Due to special properties [5], metamaterial\MSs have various applications, such as designing antennas with improved bandwidth [6–9], suppressing mutual coupling [10–12], energy harvesting [13,14], and cloaking [15]. Recently, metamaterials/MSs have been utilized as energy collectors instead of traditional antennas, exhibiting significantly higher efficiency. These metamaterials/MSs consist of arrays of small electrical resonators, such as split-ring resonators (SRR) [16] and complementary split-ring resonators (CSRR) [17,18]. Furthermore, energy collectors are typically designed in array form to capture large amounts of energy from the surrounding environment. The distance between array elements is smaller compared to conventional antenna arrays. The concept of the MS harvesters is similar to that of the MS absorbers, except that the absorbed power is dissipated in the substrate material [19–23], while in the MS harvesters, the absorbed energy is directed to the rectifier circuit. In the MS harvester design, the input impedance of each harvester is modelled by the grounded resistive load and it should be matched well to the impedance of free space to achieve higher efficiency. In some reported metamaterial/MS absorbers, the absorbed power is dissipated into resistor loads placed between the section of a resonator [24–29]. However, these loads are placed on the resonator’s surface rather than being grounded, which makes it challenging to replace them by combining networking or a rectifier circuit. The performance of the MS-based energy harvester is enhanced by various works, including improving bandwidth using an array of chaotic bow-tie CSRR [30], improving the harvesting efficiency using an array of electric-inductive capacitive (ELC) resonators [31], polarization-independent and wide-angle reception [32–35], and wide-band [36,37]. Furthermore, multi-band MS harvesters were designed to improve their functionality of harvesting the EM power from several radiation sources at various frequencies [38–42].

In the aforementioned research papers, each element of the MS harvester array is connected to one or more grounded resistive loads that mimic the rectification circuit. The harvesting efficiency of a finite array can be calculated by multiplying the capture efficiency of the central unit cell by the total number of array elements. In other words, current research into microwave energy collection with metamaterial/MS resonators focuses primarily on element design and energy capture efficiency for a single element in an array. However, the power received by each MS array element is typically less than what is required to turn on the rectifier diode. To overcome this challenge, an additional layer is added to the metasurface to develop a feeding network that gathers the power received by all array elements and delivers it to a single rectification circuit [43–48]. In [43], the MS harvester is integrated with a rectifier circuit and rectifier diodes are placed in each array unit cell to directly rectify the received EM power. However, this design approach leads to increased utilization of rectifier circuits resulting in more complicated designs, higher cost and greater energy losses.

In this study, an MS-based energy harvester is designed using an array of double-elliptical cylinder resonators. First, the design and analysis of an infinite MS structure were conducted. The proposed MS unit cell demonstrated high efficiency in capturing EM power with near-unity absorption at normal incidence. The harvesting efficiency was further improved to 94% and 72% under normal and oblique incidences respectively. Next, to increase the power density delivered to the rectification circuit and to improve activation of the rectifier diode on time, a 4x4 MS array integrated with a corporate feed network delivers the power captured by all array elements into a 50 Ω port was investigated and analysed. The proposed MS harvester can absorb EM power and deliver most of the absorbed power to the load, maximizing power harvesting efficiency.

## 2. Metasurface unit cell design

**Fig 1:**
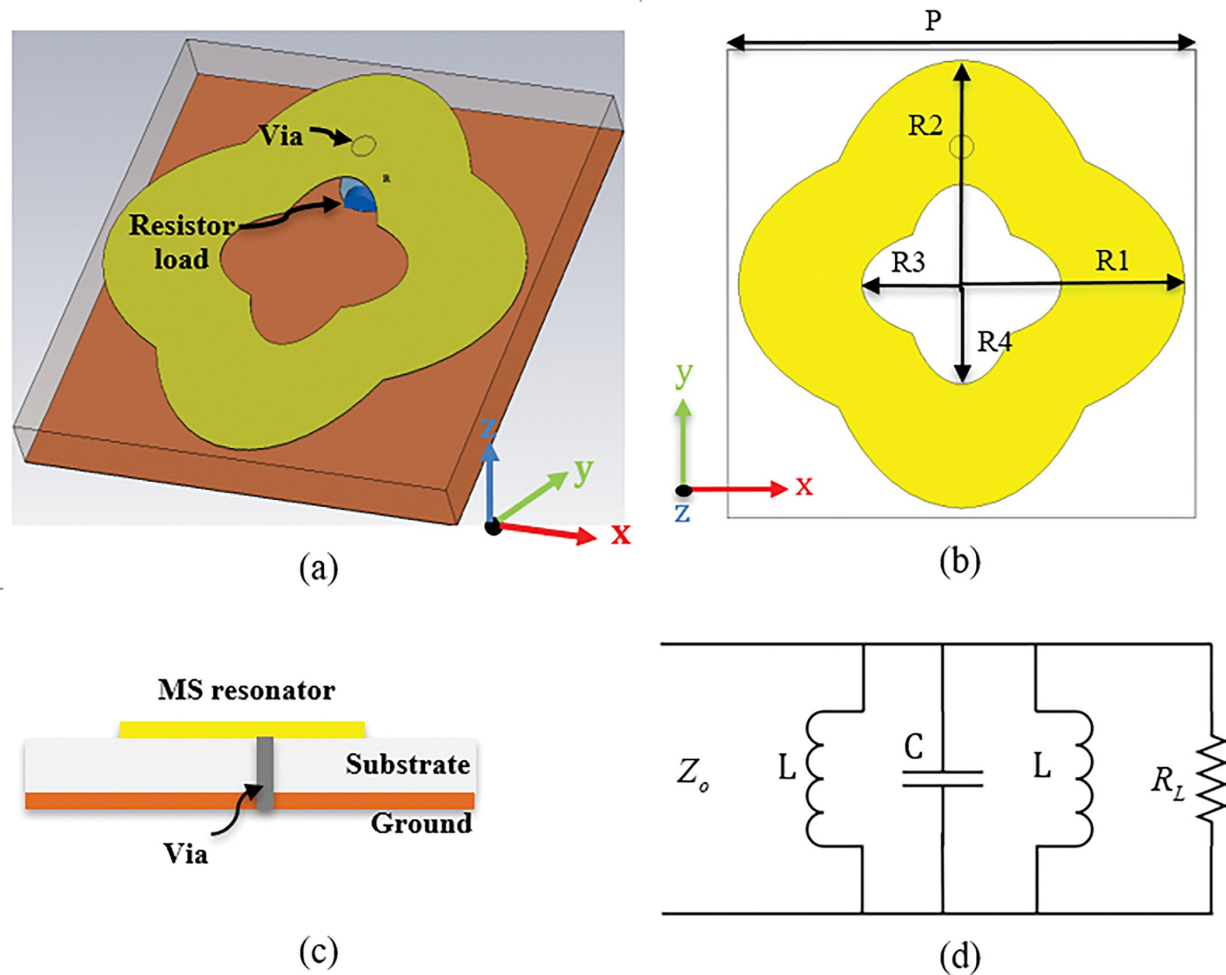
Geometry of the proposed MS unit cell (a) 3D view (b) Back view, (c) side view, and (d) equivalent circuit.

Fig 1 shows the geometry of the proposed MS harvester unit cell for energy harvesting application. The top resonating patch consists of a double elliptical resonator hosed on a 1.542 mm thickness Rogers RO4003C substrate with a dielectric constant of 3.55 and a loss tangent of 0.0027. A low-loss substrate material was chosen to reduce the impact of dielectric loss on harvesting efficiency. The bottom part of the dielectric material is completely covered with copper to block the transient. The top metallic resonator is connected with the ground plane by a resistive load through a metallic via. The proposed MS structure’s parameter details are so chosen that, at the resonance frequency of 5.3 GHz, the impedance of the resonating patch is almost equal to the impedance of free space to achieve a near unity absorption and higher harvesting efficiency. The optimized parameters of the proposed MS harvester are presented in Table 1. The optimal resistive load and via position were crucial design factors in the energy harvesting unit cell. The optimal resistance was 110 Ω, which matched well with the input impedance of the proposed MS harvester. The via was positioned at the top of the resonator to enhance the flow of the surface current from the resonator to the resistive load. The elliptical-shape resonator is chosen due to its simplicity and because it allows more efficient capture of the incident EM power due to its large aperture and wider bandwidth.

**Table 1:** Optimized dimensions of the proposed MS unit cell.

Parameter	Value (mm)
Periodicity (P)	15.7
Outer diameter of metallic elliptical cylinder (R1)	7.5
Inner diameter of metallic elliptical cylinder (R2)	4.95
Outer diameter of vacuum elliptical cylinder (R3)	3.3
Inner diameter of vacuum elliptical cylinder (R4)	1.9

To simplify the study and illustrate the energy capture mechanism, Fig 1(D) displays the equivalent circuit of the double elliptical resonator. A transmission line with characteristic impedance Z_o_ = 377 Ω is considered to be the incident plane wave. The parallel connection of the inductors and the capacitor forms the matching circuit. In the circuit, the capacitor can be constructed using the split, and the inductor can be built using the top metallic patch of the resonator and ground plane. The approximate values of the inductor and capacitors are L = 0.93 nH and C = 1.84 pF.

To examine the proposed MS structure in this study, the numerical results are performed using the FDTD method-based CST EM simulator. In the boundary condition setup, a unit cell boundary condition is applied along the x and y axes and an open boundary on the z-axis. The EM waves propagate along the ±z direction where the electric and magnetic fields are parallel and perpendicular to the x- and the y-axes, respectively.

The absorptivity of the proposed structure can be calculated using two fundamental frequency-related parameters: reflectance *R*(*ω*) and transmittance *T*(*ω*). Eq (1) is used to describe the absorbance as follows

A(ω)=1−R(ω)−T(ω) (1)

Using the scattering parameters, the reflectance and transmittance can be expressed as $R(\omega )=|S_{11}(\omega ){|}^{2}$ and $T(\omega )=|S_{21}(\omega ){|}^{2}$. Since the proposed MS harvester’s ground plane is made of copper, its transmission is given as $T(\omega )=|S_{21}(\omega ){|}^{2}\approx 0$. Then, Eq (1) can be expressed as $A(\omega )=1-R(\omega )=1-|S_{11}(\omega ){|}^{2}$ the full absorption can be achieved by the minimal amount of the reflection coefficient which can be achieved by a good match between the proposed MS harvester’s input impedance and that of the free space.

## 3. Results and discussion

**Fig 2:**
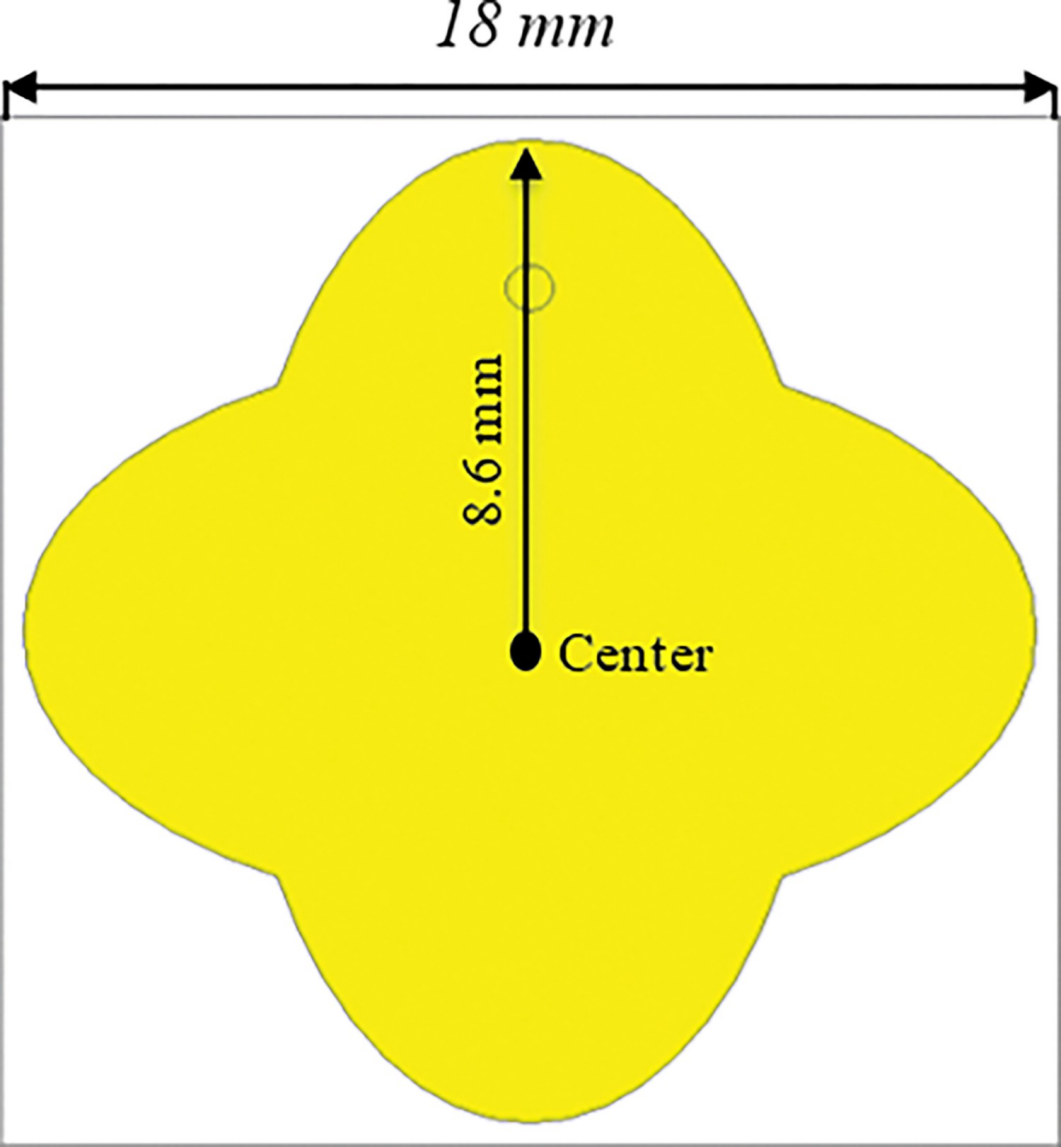
Schematic of the proposed MS harvester unit cell.

The performance of the proposed MS unit cell harvester was optimized by evaluating the geometry of the unit cell design. Fig 2 shows the proposed MS harvester with overall dimensions of 18 mm × 18 mm and an 8.6 mm diameter of the elliptical cylinder.

**Fig 3:**
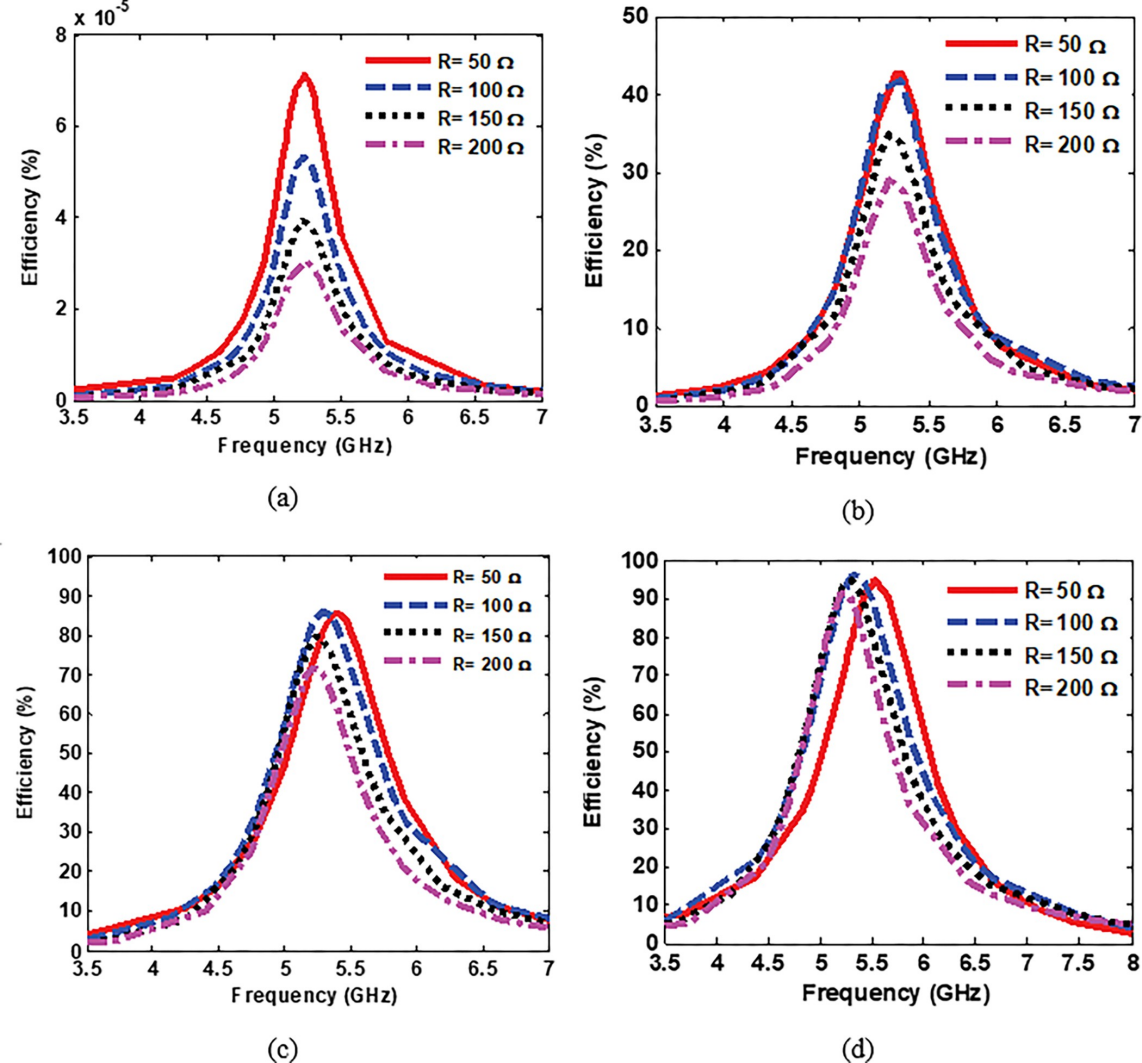
Numerical results demonstrating the efficiency of the proposed MS harvester with terminated loads ranging from 50 Ω to 200 Ω, and having a via positions distance from the resonator’s centre of (a) centre, (b) 2 mm, (c) 4 mm and (d) 6 mm.

First, in the simulation setup, to investigate the effects of the via-hole position away from the centre of the resonator in terms of the harvesting efficiency, the via-hole position away from the centre of the resonator was varied from 0 mm to 6 mm. In all four cases of the via hole positions away from the centre, the resistive loads are swept from 50 Ω to 200 Ω. Then, the harvesting efficiency of the proposed MS harvester was computed. Fig 3 shows the numerical results of the harvesting efficiency of the proposed MS harvester at different via-hole positions away from the resonator. When the via hole is positioned at the centre of the resonator, the harvesting efficiency is too low (almost neglected). By moving the via hole up to 6 mm away from the resonator’s centre, peak efficiency about 96% is achieved when the MS structure is terminated by a load resistance of 100 Ω as can be seen in Fig 3(D). Furthermore, at the position 6 mm away from the resonator’s centre, the proposed MS harvester can efficiently capture the EM power with a wide range of the terminated load resistances from 50 Ω to 200 Ω with efficiencies exceeding 88% as shown in Fig 3(D).

### 3.1 MS unit cell harvester integrated with slots

**Fig 4:**
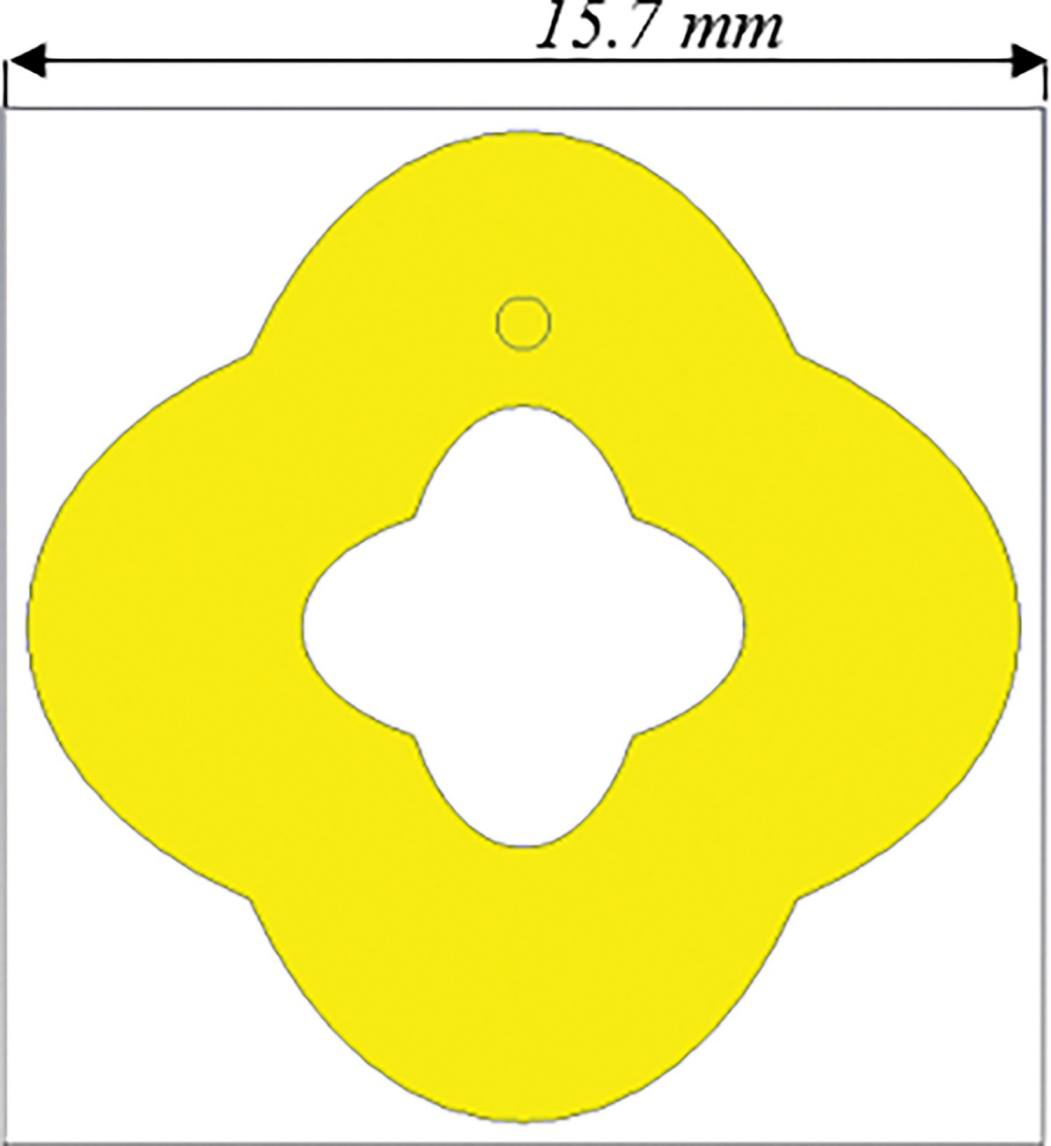
Geometry of the proposed MS harvester integrated with slots.

Fig 4 shows the geometry of the proposed MS unit cell harvester integrated with slots. It is noted that the size of the MS unit cell structure is reduced compared to the MS structure in Fig 2. Subsequently, it is numerically investigated as an infinite unit cell structures to demonstrate its performance in terms of EM power absorption and its delivery to the load.

**Fig 5:**
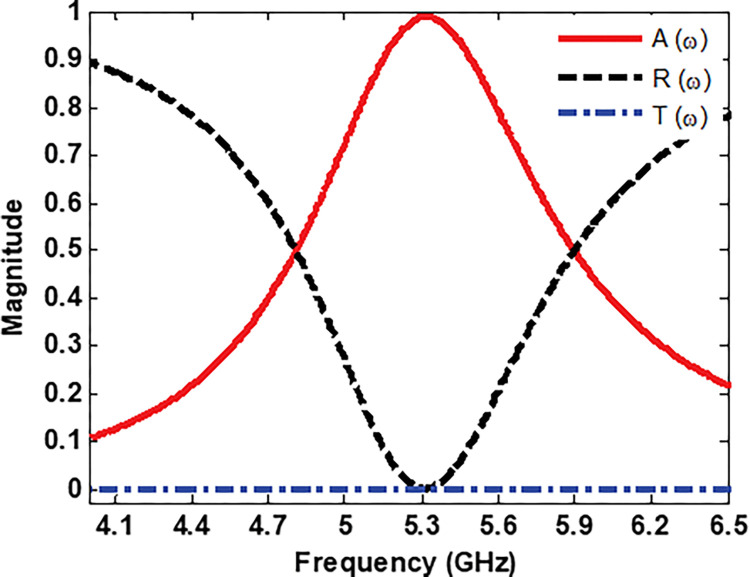
Absorption, reflection and transmission coefficients.

The numerical coefficients of the absorption, reflection and transmission are shown in Fig 5. From Fig 5, it can be seen that the maximum absorption peak is more than 99% at 5.3 GHz. Furthermore, the fractional bandwidth (FBW) of the proposed MS unit cell harvester is calculated as [49]

FBW=Δffc (2)

where Δ*f* and *f*_*c*_ are the half-power bandwidth and centre frequency respectively. For the proposed MS harvester, these parameters, as obtained in the simulation, are Δf = 1.11 GHz, f_c_ = 5.3 GHz and *FBW*≈21%.

**Fig 6:**
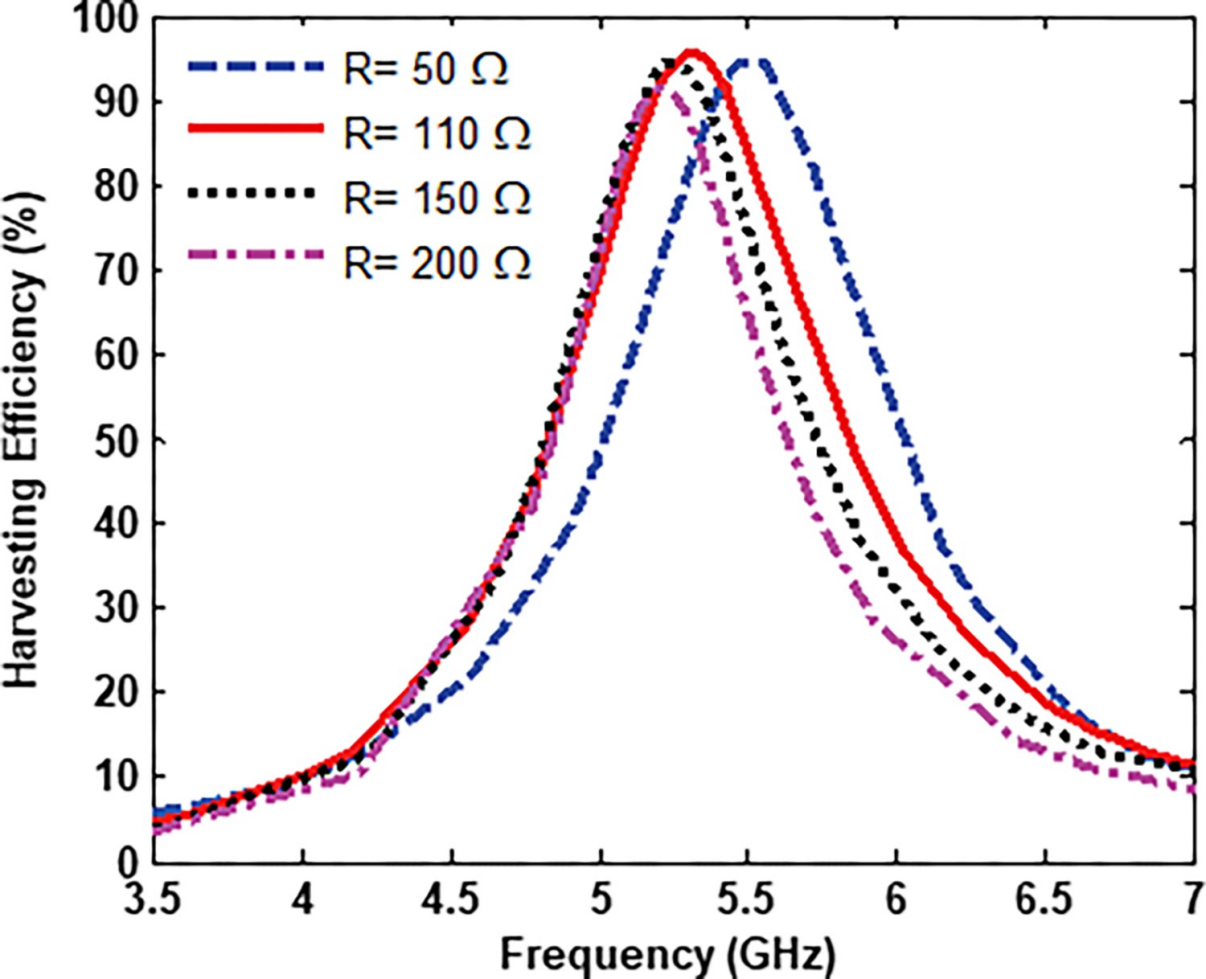
Harvesting efficiency at different resistor loads.

A resistive load is a critical factor in the design of the EM energy harvester. The harvesting efficiency is investigated when the terminated resistive loads are swept over the range from 50 Ω to 200 Ω, as shown in Fig 6. The efficiency of about 95% is achieved when a resistor load of 110 Ω terminates the unit cell which is almost equal to the input impedance of the proposed MS unit cell harvester. Furthermore, the proposed MS unit cell harvester can capture EM power over the terminated resistor loads ranging from 50 Ω to 200 Ω, exceeding harvesting efficiency of about 88% at 5.3 GHz, as shown in Fig 6. This makes it simple to construct a corporate feed network to connect all the array elements into a single load.

**Fig 7:**
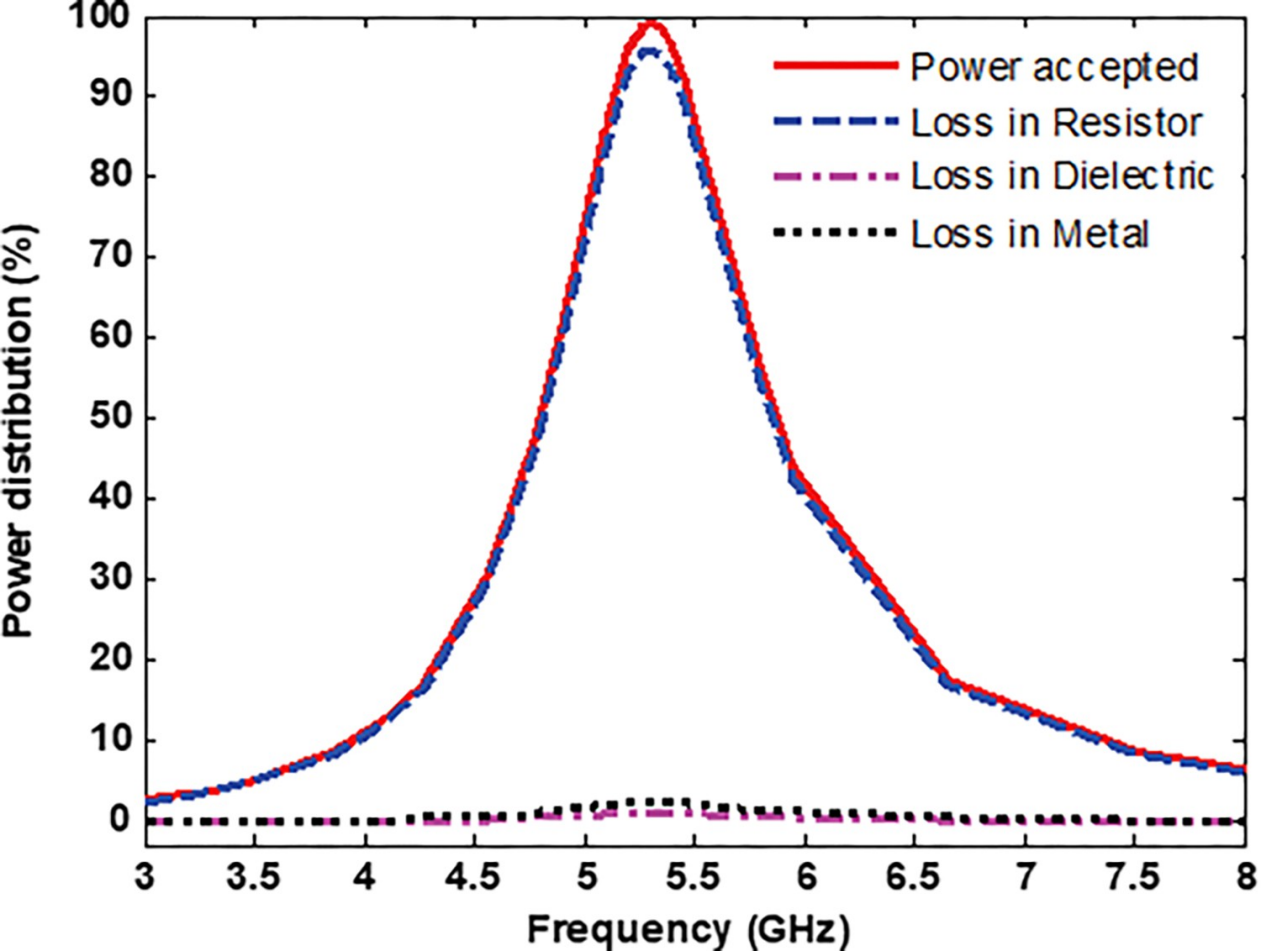
Power distribution into the MS unit cell.

Next, the dissipated power distribution into the MS unit cell structure is analysed. Fig 7 shows the total power absorbed by the MS unit cell and harvesting power efficiency in the resistor load, dielectric substrate (Rogers material) and metal when the incident electric field (E-field) propagates along the y-axis. The radiation -to- AC efficiency of the MS harvester is defined as

$$\eta_{rad\_AC}=\frac{P_{load}}{P_{incident}}$$ (3)

where *P*_*load*_ is the total average power dissipated in the load and *P*_*incident*_ is the total average power incident on the MS footprint. As shown in Fig 7, most of the power absorbed by the proposed MS harvester is mainly concentrated in the resistor load achieving 95% at the resonance frequency of 5.3 GHz. While the power dissipated in the dielectric and metal is neglected. The high power dissipated in the resistor load can be attributed to some factors such as the low-loss dielectric substrate (Rogers RO4003C), effective impedance matching between the MS structure and free space, and optimal positioning of the vias.

**Fig 8:**
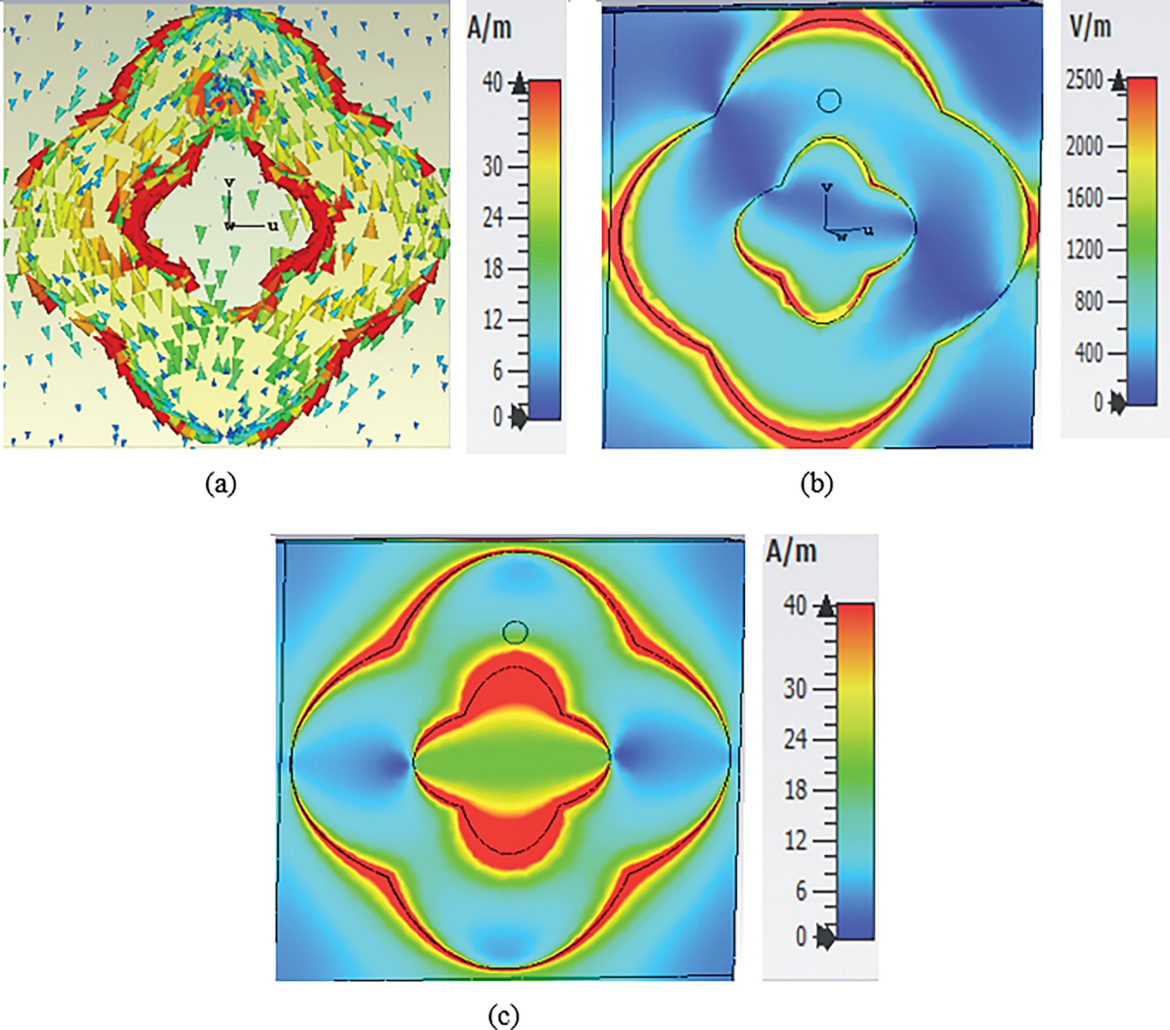
Simulation results (a)surface current (b) E-field and (c) H-field distributions at 5.3 GHz for the MS unit cell.

To better understand the electrical properties of the proposed MS unit cell harvester, the surface current, electric field (E-field) and magnetic field (H-field) distributions were calculated and plotted in Fig 8. As can be seen in Fig 8(A), the current flow is anti-parallel. Furthermore, a high-intensity current was observed to be induced within the double-elliptical cylinder resonator, particularly in the middle and was then delivered to the resistor load through a metallic via to achieve a near-unity absorption. The intensity of the E-field is more concentrated near the outer edges than the middle, as shown in Fig 8(B). This effect is caused by the strong mutual coupling between the adjacent top layers of the unit cells. To achieve maximum absorption, electric and magnetic resonances must occur simultaneously. Fig 8(C) presents the H-field at 5.3 GHz, where a strong field appears at the outer edges of the resonator and the middle as well.

**Fig 9:**
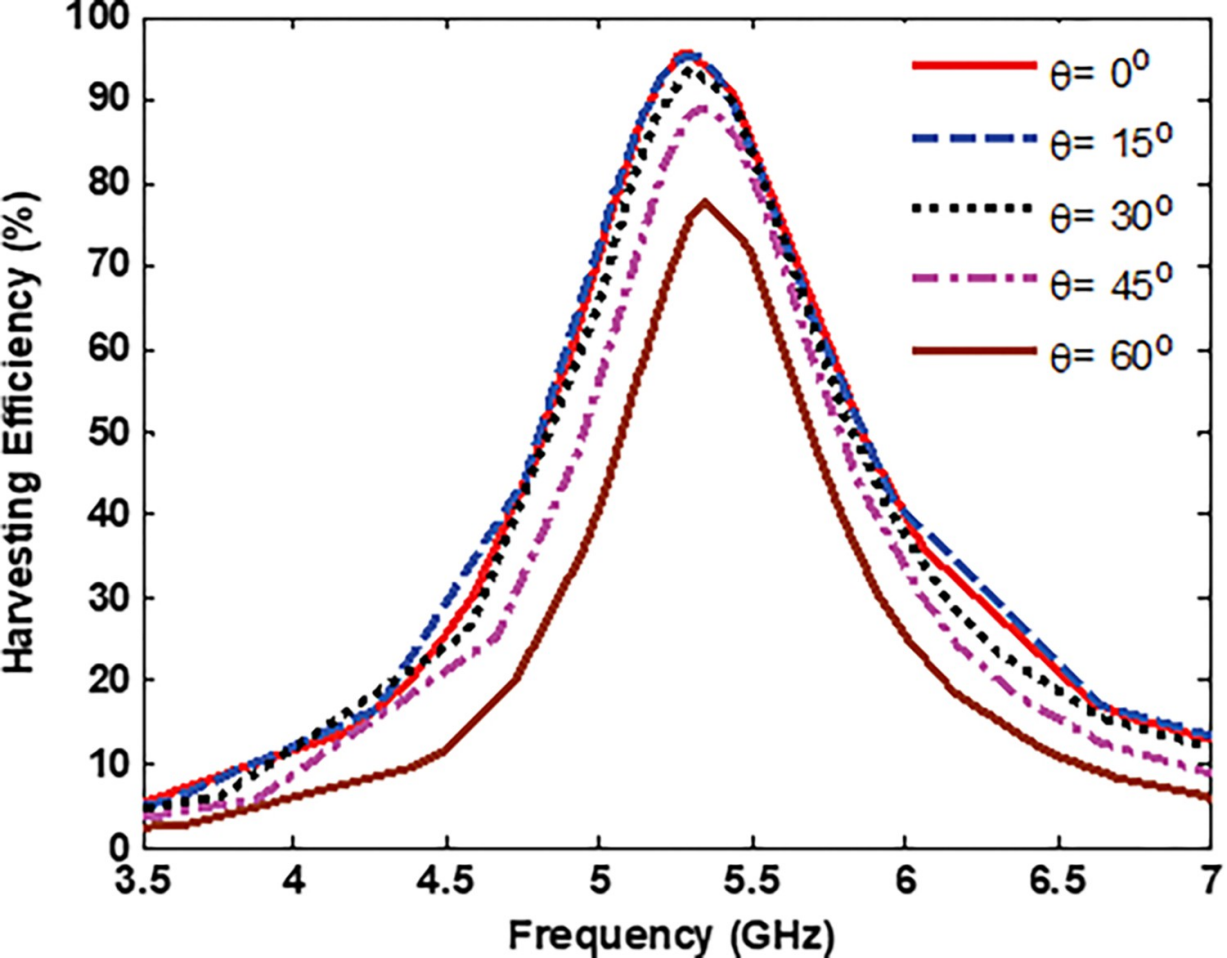
Unit cell efficiency vs frequency for different incident angles.

A comprehensive analysis of the loss balance in the load was conducted to evaluate the energy harvesting capabilities of the proposed MS unit cell. The ratio of the total power delivered to the load to the power incident on the unit cell surface was calculated for different incident angles of the EM wave under TE polarization. The power-harvesting efficiency of the MS unit cell was calculated numerically and is depicted in Fig 9, covering incident angles from 0^*o*^ to 60^*o*^ in 15^*o*^ increments. As can be seen in Fig 9, the harvesting efficiency of about 95% is achieved at normal incidence. For oblique incidence, it remained above 84% for incident angles up to 45^*o*^ and 74.2% for an incident angle of 60^*o*^.

### 3.2 4 × 4 metasurface array with corporate feed network

**Fig 10:**
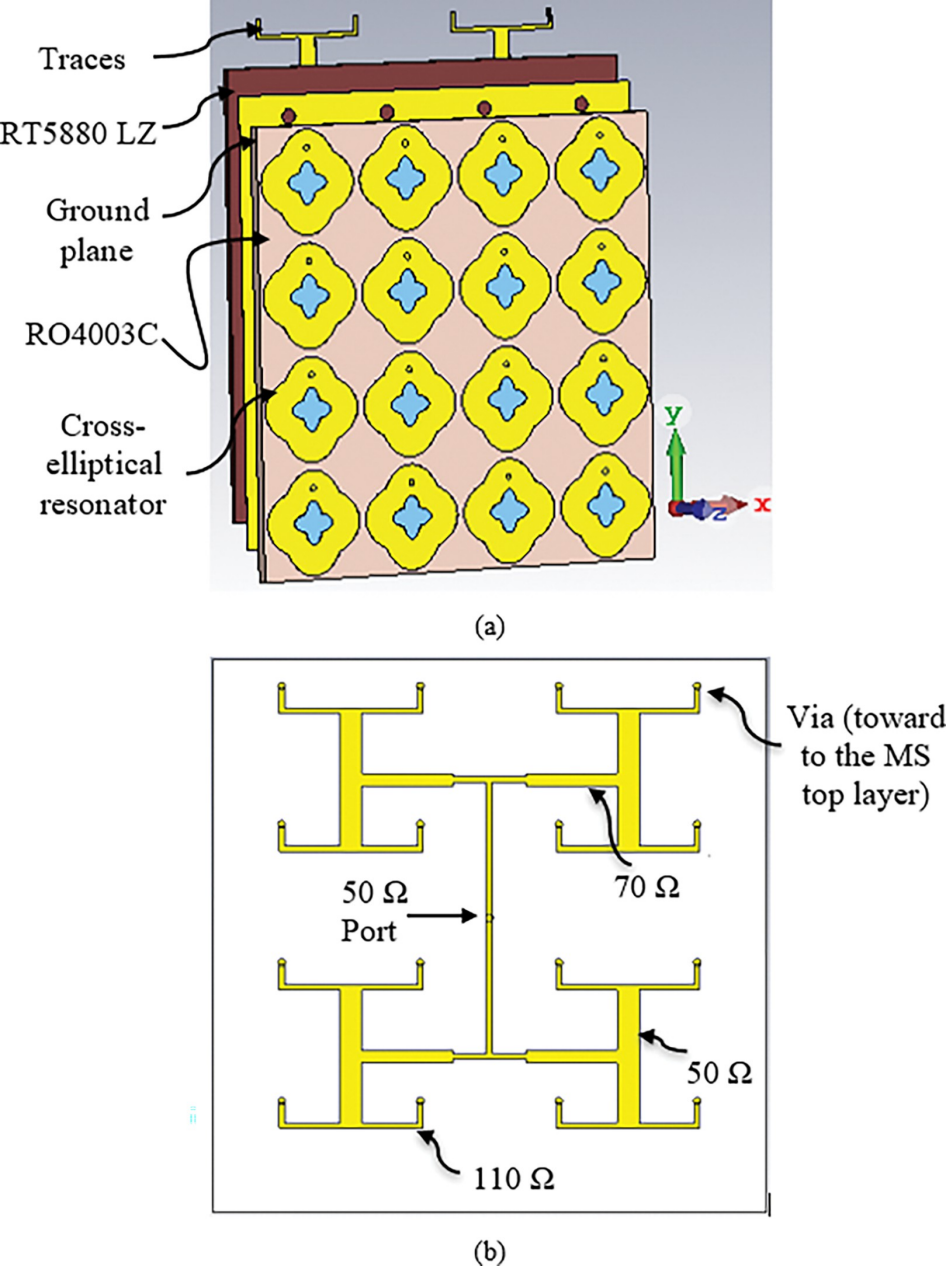
(a) Schematic of the metasurface harvester is shown as an exploded view, including a cross-elliptical resonator, Rogers RO4003C as the first substrate, a ground plane (copper), Rogers RT5880LZ as the second substrate, and the transmission traces, and (b) Configuration of corporate feed network.

The proposed unit cell demonstrated the harvester’s potential to capture ambient EM power and transfer it to a resistive load. However, the power received by a single MS unit cell is insufficient to operate even small devices. Thus, an array of MS elements is required to provide sufficient power to a device or system. In this section, a 4x4 array of MS resonators with overall dimensions of 62.8 mm x 62.8 mm was designed and analysed to achieve high power-harvesting efficiency, as depicted in Fig 10. The array elements were all connected to a single 50 Ω port through a corporate feed network. In energy harvesting designs, the spacing between neighbouring unit cells is crucial, as adjusting it can impact the MS unit cell impedance. A small periodicity spacing is often desirable, leading to a high coupling between array elements and increased received power. However, this small periodicity spacing requires the use of an additional substrate under the ground plane for constructing the feed network, which is intended to direct the array’s total energy to a single resistive load. Rogers RT5880LZ substrate with a dielectric constant of *ε*_*r*_ = 2, a loss tangent of *δ* = 0.0021, and 1.2 mm thickness was attached underneath the ground plane to handle the microstrip line traces. The corporate feed network connects all MS array elements to a single 50 Ω port, which was chosen to match most measurement devices and thus eliminate the need for matching circuits during measurement. The corporate feed network is designed using the same technique used in the antenna corporate feed network. The length and width of the traces can be calculated using the following equations [50]

$$W=d\frac{8e^{A}}{e^{2}A-2}\;for\;\frac{W}{d}<2$$ (4)

$$\begin{array}{c} W=d\frac{2}{\pi }[B-1-\ln(2B-1)+\frac{\varepsilon_{r}-1}{2\varepsilon_{r}}(\ln(B-1)+0.39-\frac{0.61}{\varepsilon_{r}}]\\ for\;\frac{W}{d}>2 \end{array}$$ (5)

where

$$A=\frac{Z_{0}}{60}\sqrt{\frac{\varepsilon_{r}+1}{2}}+\frac{\varepsilon_{r}-1}{\varepsilon_{r}+1}(0.23+\frac{0.11}{\varepsilon_{r}})$$ (6)

and

$$B=\frac{377\pi }{2Z_{0}\sqrt{\varepsilon_{r}}}$$ (7)

*ε*_*r*_ is the dielectric constant, *d* is the substrate thickness, *Z*_0_ is the characteristic impedance, and *W* is the microstrip line width. The guided wavelength is

$$\lambda_{g}=\frac{\lambda_{0}}{\sqrt{\varepsilon_{re}}}\;or\;\lambda_{g}=\frac{300}{f(GHz)\sqrt{\varepsilon_{re}}}$$ (8)

where *λ*_0_ is the free space wavelength and *ε*_*re*_ is the effective dielectric constant which given by

$$\varepsilon_{re}=\frac{\varepsilon_{r}+1}{2}+\frac{\varepsilon_{r}-1}{2}\frac{1}{\sqrt{1+12\frac{d}{W}}}$$ (9)

Three different transmission lines were used to design the routing, optimized to achieve a better reflection coefficient and higher power efficiency. The optimized widths of the transmission lines were 2.38 mm, 1.36 mm, and 0.64 mm for 50 Ω, 70 Ω and 110 Ω respectively. Fig 10(B) shows the configuration of the corporate feed network for the metasurface harvester array.

## 4. Measurement results and discussion

**Fig 11:**
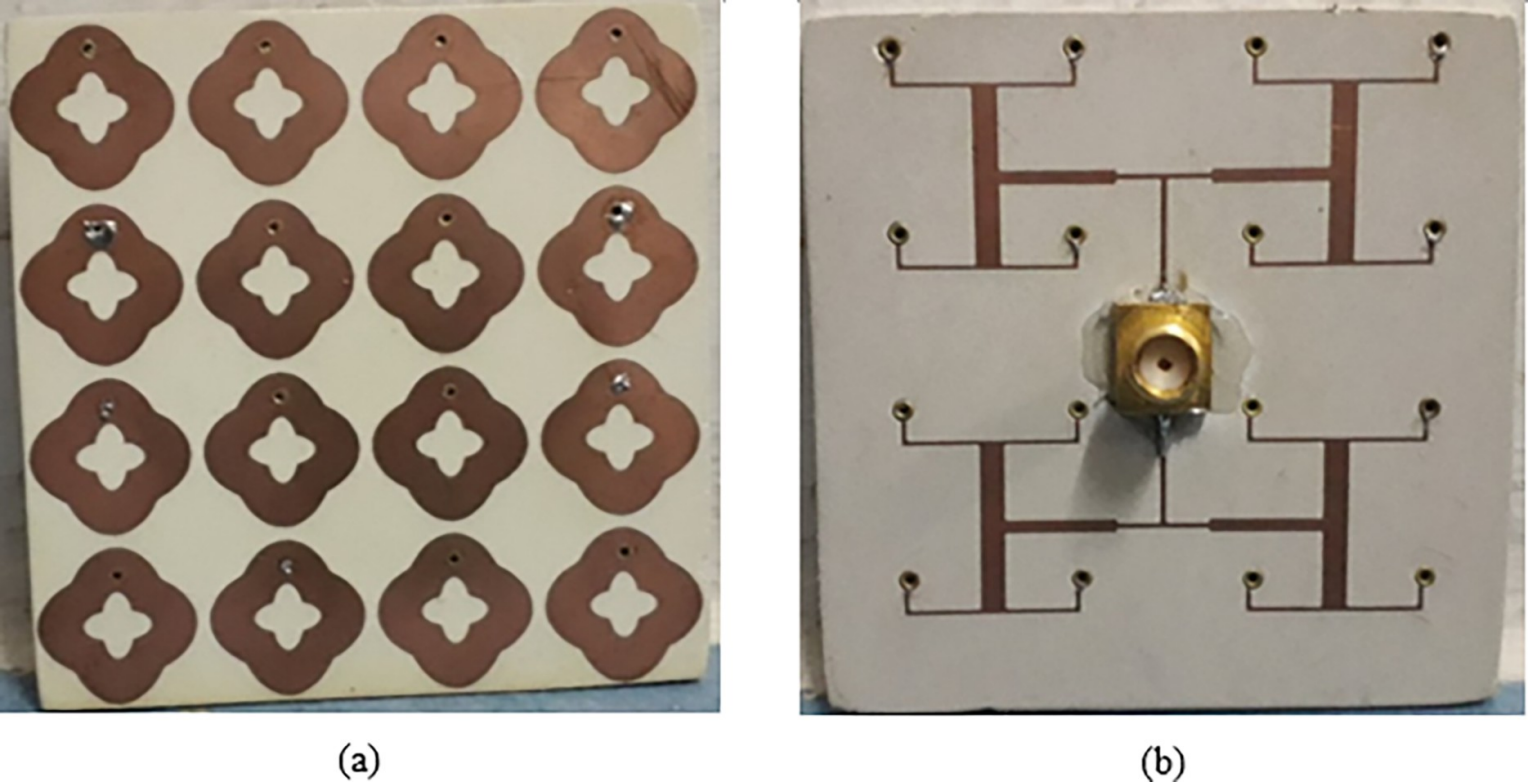
Fabricated metasurface array (a) Elliptical resonators and (b) Corporate feed network.

In light of the obtained numerical results for the MS energy harvester with a corporate feed network, a finite MS harvester array consisting of 4 x 4 double elliptical resonator elements was fabricated using a printed circuit board, as depicted in Fig 11. The MS resonators were printed on a Rogers RO4003C as shown in Fig 11(A). The corporate feed network is hosted on the Rogers RT5880LZ substrate to connect all the MS array resonators to one 50 Ω load as shown in Fig 11(B). Each resonator of the MS array is connected with corporate feed lines through a metallic via. The SAM connector is connected the ground plane with the corporate feed network.

**Fig 12:**
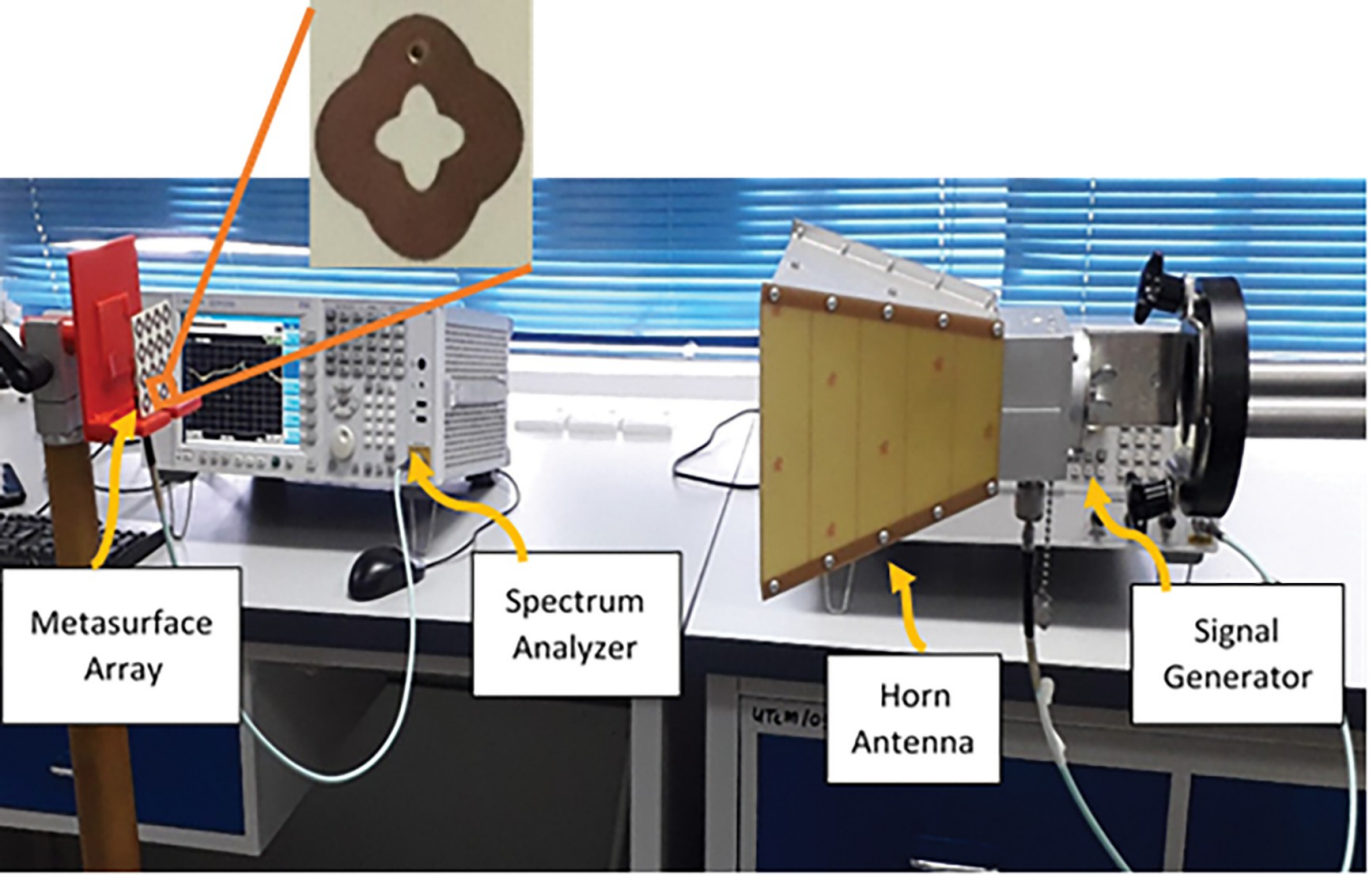
Efficiency measurement setup for the MS array harvester.

To evaluate the radiation-to-AC efficiency of the proposed MS array in terms of the received power, an experiment was carried out, as depicted in Fig 12. The experiment utilized an RF signal generator set at 3 dBm, which transmitted a signal to the horn antenna (type HF906) within the designated measurement band. The fabricated MS array was positioned in the far-field region of the transmitting horn antenna and was connected to a spectrum analyser for measuring the received power. To ensure that the MS harvester is effectively stimulated by a plane wave in the far-field region, the appropriate distance between the transmitter antenna and the MS harvester sample can be calculated as follows [51]

$$R>\frac{2D^{2}}{\lambda }$$ (10)

where *R* is the distance between horn antenna and harvester sample, *D* is the largest antenna’s aperture, and *λ* is the wavelength.

The overall harvesting efficiency can be described by the Eq 3. The power incident into the MS footprint *P*_*incident*_ can be defined as follows

$$P_{incident}=\frac{G_{t}.P_{t}}{4\pi R^{2}}A_{array}$$ (11)

where *G*_*t*_ is the horn antenna’s gain, *P*_*t*_ represents the power excited by the signal generator, *R* is the distance between the horn antenna and fabricated MS array which is R = 31 cm, and *A*_*array*_ is the effective area of the MS array.

**Fig 13:**
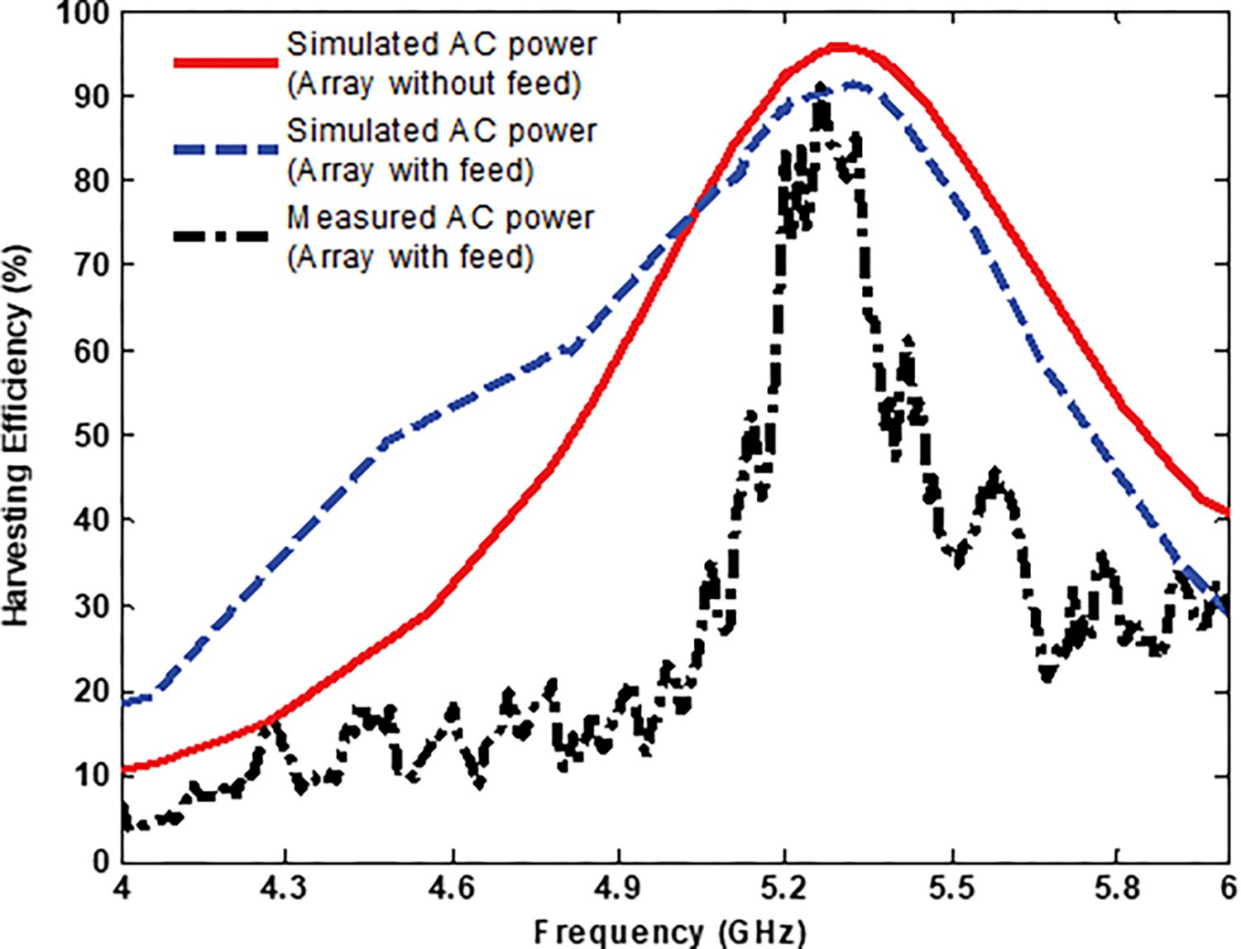
Simulated and measured AC power efficiency.

Fig 13 shows the radiation-to-AC efficiency of the MS array obtained from the simulation and the measurement under normal incidence. The measured peak efficiency of radiation-to-AC efficiency was 90%, while the simulation yielded 94%. The shift in the centre frequency is caused due to the fabrication tolerance and measurement environment. Accounting for practical physical mechanisms of loss that were not included in the simulation, a difference of 4% between measured and simulated peak efficiencies is considered acceptable.

Table 1 presents a summary of the performance and characteristics of the proposed MS unit cell harvester, in comparison to previously published works. It should be noted that the proposed MS unit cell exhibits a smaller size and higher harvesting efficiency compared to previously published research works [33,36,53]. While the works presented in [30,52] have smaller sizes, their harvesting efficiency is lower than that achieved by the proposed MS harvester. Additionally, the proposed harvester achieves a remarkable efficiency relative bandwidth of 4.2% with a significant 90% efficiency. To summarize, this research introduces notable innovations when compared to aforementioned references listed in Table 2, including a higher harvesting efficiency of approximately 95% and a substantial 90% efficiency relative bandwidth of 4.2%.

**Table 2:** Comparison of the MS unit cell’s AC efficiency and 90%—efficiency relative bandwidth with other published research.

Ref. Year	Structure	Size of structure	Freq. (GHz)	Substrate Material	AC Efficiency	90%—efficiency relative bandwidth
[30]	CSRR	0.22*λ*_*o*_	6.1	Rogers RO4003	87%	N/A
[33]	Rotating central symmetry	0.32*λ*_*o*_	5.8	-	88%	N/A
[36]	square ring resonator	0.48*λ*_*o*_	15	F4B	90%	>1%
[52]	Electric ring resonator (ERR)	0.22*λ*_*o*_	5.54	RO4003C	91%	>1%
[53]	Symmetrical circular sectors	0.29*λ*_*o*_	5.8	-	91%	>1%
This work	Double elliptical resonator	0.27*λ*_*o*_	5.3	RO4003C	95%	4.2%

## 5. Conclusion

This work presents an MS energy harvester that can efficiently capture EM waves and deliver the absorbed power into a single resistive load. To begin, the proposed MS unit cell was modelled as an infinite array using periodic boundary conditions. Simulations revealed a higher absorption of about 90% across the frequency range from 5.15 GHz to 5.5 GHz, and fractional bandwidth is 21%. Furthermore, the proposed MS unit cell harvester can efficiently capture the incident EM waves with a wider oblique incident angle of up to 60^*o*^. Then, a 4x4 MS array with a corporate feed network was designed and fabricated. The corporate feed network is used to connect all the elements of the array to a single 50 Ω load to improve the radiation to AC efficiency. Both numerical full-wave analysis and experimental measurements were performed to assess the performance of the proposed MS harvester and the results indicated an overall harvesting efficiency of about 90%. Due to its higher efficiency, the proposed MS array can be a good candidate for EM energy harvesting applications.

## Supporting information

S1 Dataset (RAR)
